# Report on the 13th international workshop on the CCN family of genes and inaugural ARBIOCOM conference, Nice, France

**DOI:** 10.1002/ccs3.70096

**Published:** 2026-08-06

**Authors:** Bernard Perbal, Ralf Weiskirchen, Annick Perbal, Brahim Chaqour

**Affiliations:** ^1^ International CCN Society (ICCNS) Nice France; ^2^ Institute of Molecular Pathobiochemistry RWTH University Hospital Aachen Aachen Germany; ^3^ Department of Molecular Ophthalmology University of Pennsylvania Perelman School of Medicine Philadelphia Pennsylvania USA

**Keywords:** association for research on biosignaling and communication (ARBIOCOM), CCN1‐6, gene expression, inflammation, international CCN society (ICCNS), international workshop on the CCN family of genes, musculoskeletal biology, normal and pathological development, tumor biology

## Abstract

The 13th International Workshop on the CCN Family of Genes, held in Nice as part of the inaugural ARBIOCOM World Conference, brought together investigators with diverse research backgrounds, disciplines, and expertise. Building on the long‐standing mission of the International CCN Society and the expanding ARBIOCOM initiative, the meeting highlighted the growing importance of interdisciplinary exchange in accelerating discovery in normal and pathological signaling networks. Scientific sessions featured presentations by renowned speakers and early‐career investigators alike, creating a highly collaborative environment that fostered discussion of emerging concepts and translational opportunities. Among the major themes, this joint conference‐meeting highlighted “hot” research topics such as the nuclear and extracellular roles of CCN proteins in regulating gene expression, tissue development and remodeling, stem cell differentiation and therapies, and disease initiation and progression. Presentations on skeletal biology emphasized how extracellular matrix proteins and matricellular factors shape bone, cartilage, and connective tissue development and pathology, whereas vascular sessions revealed new mechanistic links between extracellular cues, endothelial remodeling, macrophage‐driven vessel regression and vascular smooth muscle homeostasis. Other talks extended the relevance of new signaling molecules in intestinal regeneration, hepatic inflammation, metabolic control, and cancer progression, illustrating the broad biological reach of these pathways. The meeting also emphasized the value of integrating new model systems, advanced single‐cell approaches, and functional in vivo studies to uncover mechanisms that were previously unknown. Collectively, the scientific sessions and discussions identified and filled key knowledge gaps and generated new hypotheses with strong potential for future discoveries. By connecting established expertise with complementary scientific perspectives, this meeting provided a fertile platform for cross‐pollination of ideas and highlighted several avenues for future research aimed at developing novel diagnostic and therapeutic strategies and tools.

## Graphical abstract

Celebration of the 13th ICCNS meeting joint to the 1^st^ ARBIOCOM Conference held on the site of Nice in December 2025.

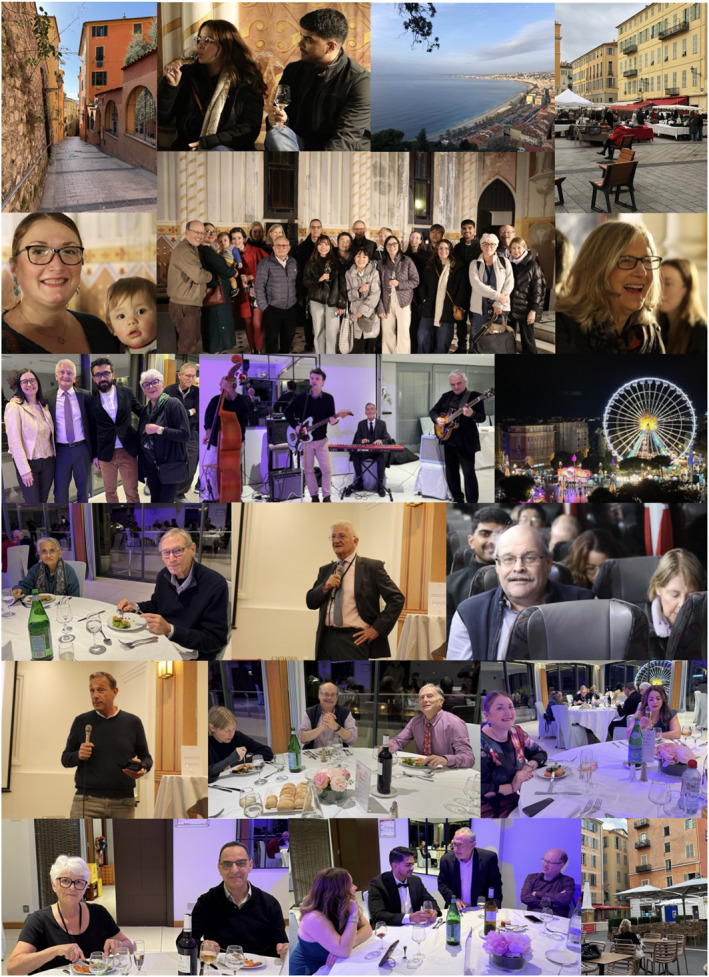

## 1 INTRODUCTION

**FIGURE 1:**
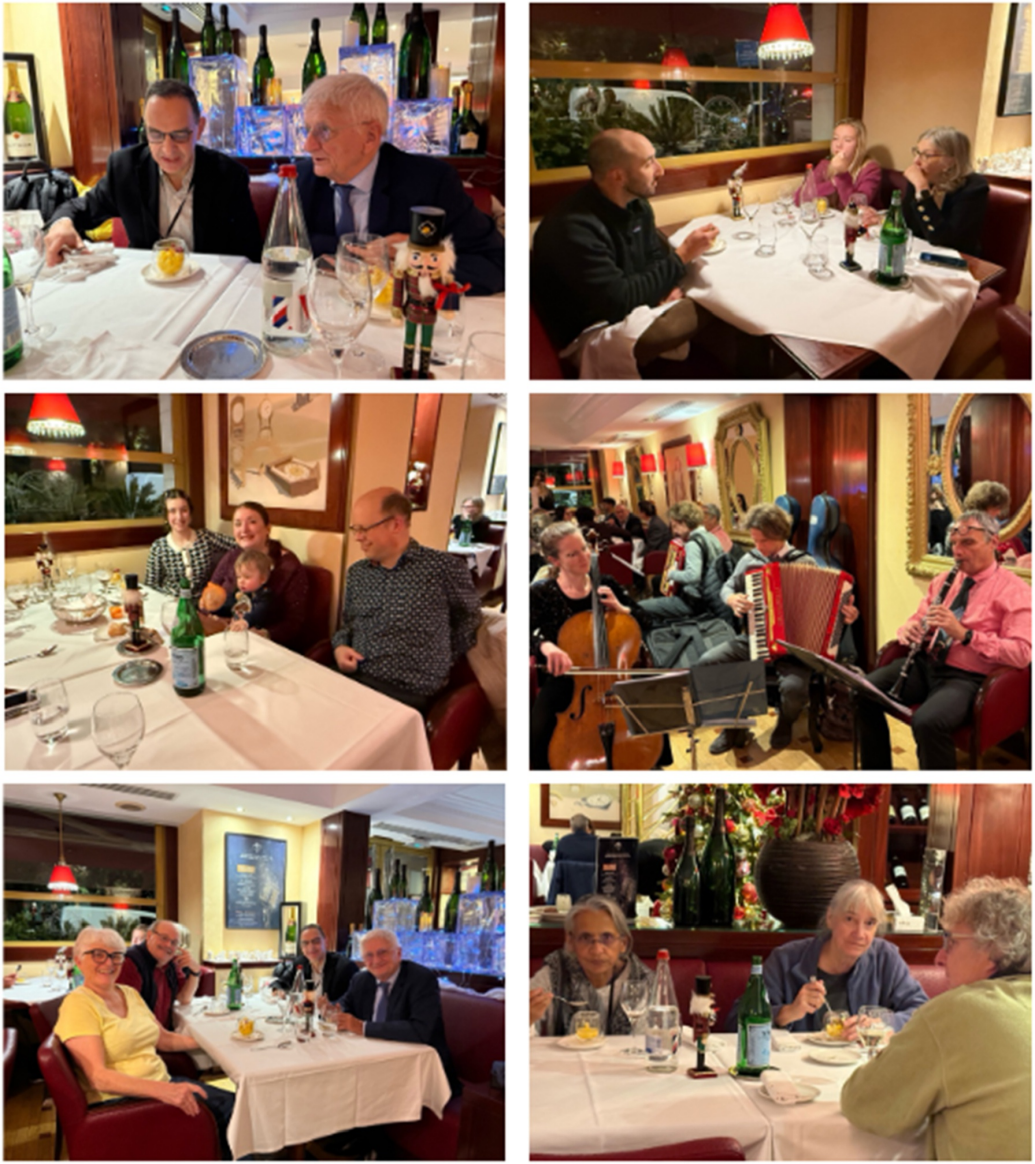
Since the first international workshop on the CCN Family of Genes, the ICCNS has organized a variety of social events for meeting participants, fostering scientific exchange and collegial interactions in a friendly atmosphere. The opening of the joint ICCNS‐ARBIOCOM meeting was celebrated with a gala dinner at the Aston Hotel, featuring a performance by a local musical group that highlighted the cultural spirit of the region.

**FIGURE 2:**
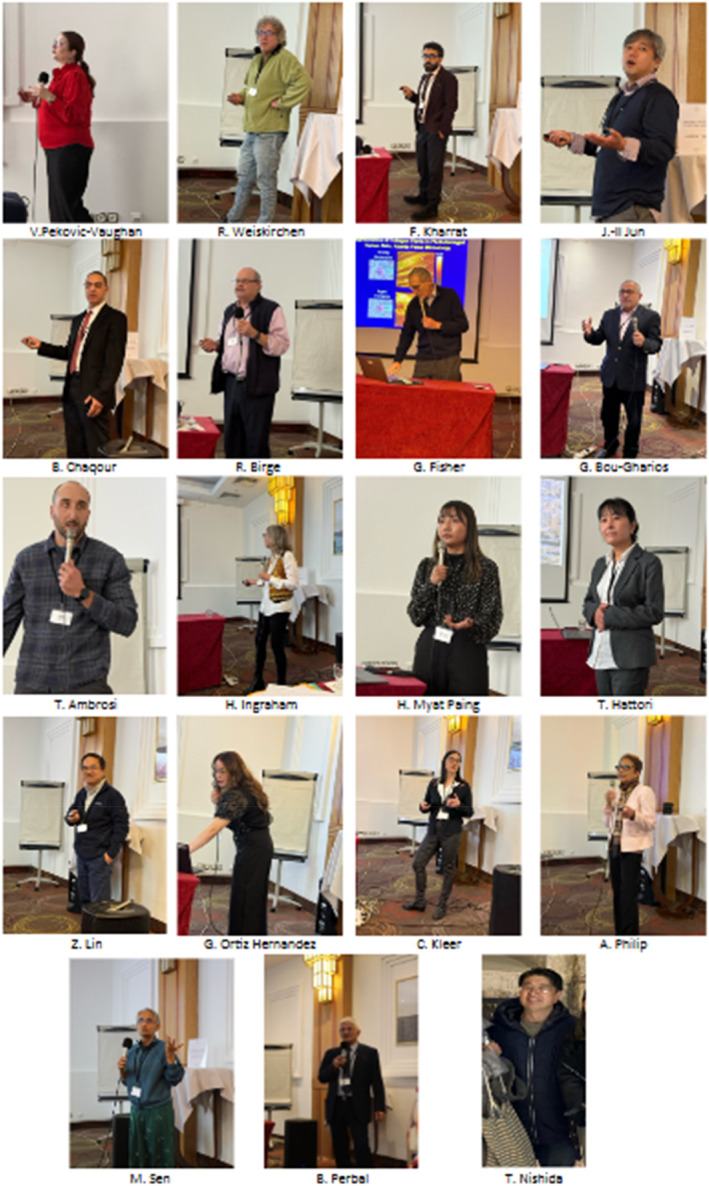
The diversity and international background of the speakers and participants contributed to a highly stimulating and productive series of scientific sessions, promoting interdisciplinary discussions, and new collaborative opportunities within the CCN research community.

The 13th International Workshop on the CCN Family of Genes, originally scheduled to take place in Chicago in June 2026 and postponed due to local logistical challenges and worldwide geopolitical instability, was held on December 13–17 as part of the Nice Inaugural ARBIOCOM World Conference. This joint event was the realization of a project initially launched at the 10th International CCN Society (ICCNS) Workshop in Niagara and later concretized through the creation of ARBIOCOM in 2020. The organization of ARBIOCOM was defined during several meetings of the executive board as a platform whose main goal is to bring together members of various scientific and medical organizations eager to exchange and share their knowledge and ideas in fields related to cell communication in a friendly and familial atmosphere (Figure 1). As a member of ARBIOCOM, the ICCNS proposed to take charge of the first joint inaugural meeting in Nice and to promote the international development and federation of biomedical organizations interested in the dissemination of both scientific and medical knowledge in the fields of normal and pathological biological signaling and communication. The diversity of attendance (Figure 2) reflected the significant progression of the ARBIOCOM establishment. The ratio of external to CCN talks was approximately 50%. The strong support of highly skilled participants from the CCN field, combined with the participation of accomplished experts in diverse complementary approaches, resulted in an extremely productive melting pot that paved the way for a better understanding of cellular signaling and for new collaborative projects. The progress fueled by new CCN results obtained along the main research streams, together with the use of previously unexplored model systems, should lead to breakthrough translational applications.

**FIGURE 3:**
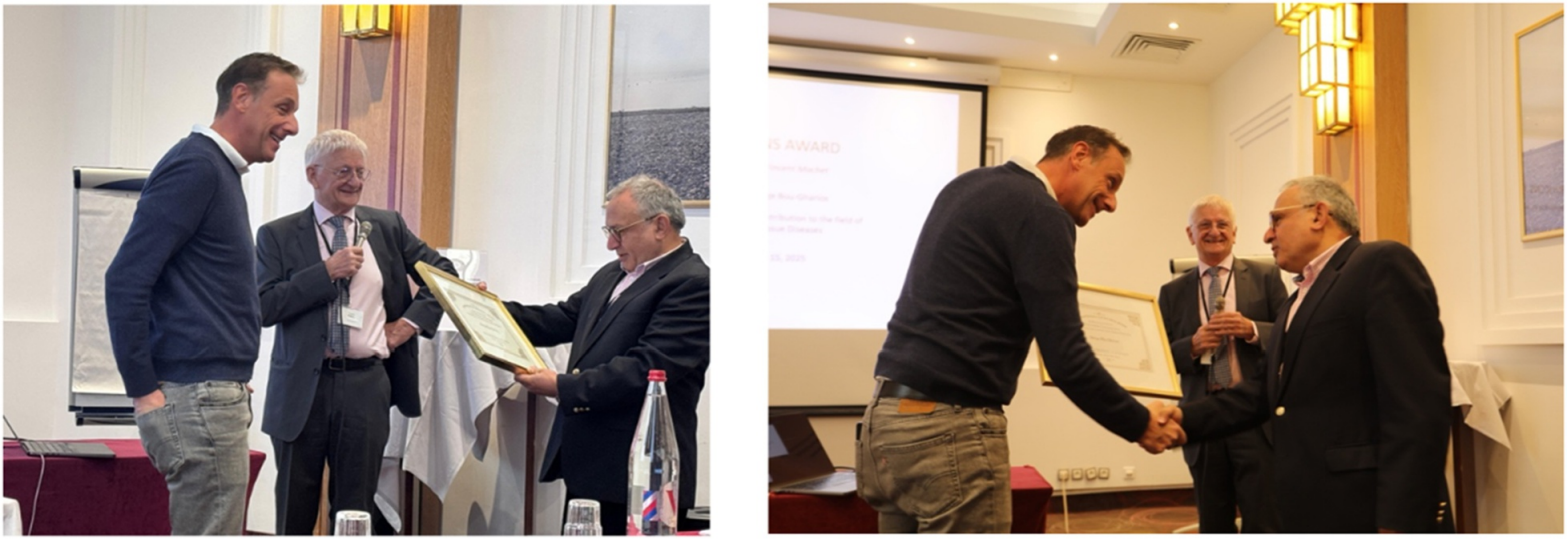
Professor George Bou‐Gharios was presented with the 10th ICCNS Award by Mr. Vincent Machet, Director of a family‐owned mid‐sized company, in recognition of his outstanding contributions to CCN research and to the activities of the ICCNS community.

In an effort to broaden the scientific scope of the International Workshop on the CCN Family of Genes, Professor Bernard Perbal, President of the ICCNS, and Peter Butler, former Editor at Springer Nature, agreed in 2008 to create an ICCNS Award to be presented to a scientist in recognition of their scientific merit and contribution to their field of interest. The ICCNS Award was intended to promote interactions between scientists working in the CCN field and those involved in related biological and biomedical areas. The 10th ICCNS Award was presented to Professor George Bou‐Gharios in recognition of his scientific merit and his contribution to the development of animal models for connective tissue disease. Studies performed using these experimental models open new avenues for therapeutic intervention in fibrosis and osteoarthritis. This award was sponsored through a personal donation from M. Vincent Machet, who covered the full cost of the meeting registration fees and the honorary plaque presented to the recipient (Figure 3).

The scientific sessions featured presentations by renowned speakers from diverse geographical regions on diverse research studies directly or indirectly involving cell communication and signaling. Six scientific sessions covered research areas in CCN proteins in dynamic biological contexts, extracellular matrix protein biology, inflammation, development, musculoskeletal biology, and tumor biology. The abstracts of all talks presented at the meeting are appended at the end of this report. For the first time in the history of the ICCNS report, we unfortunately experienced last‐minute abstract withdrawals from French and American colleagues who had previously committed to delivering oral presentations and chairing sessions.

We have chosen to highlight below four topics that stood out during the meeting, reflecting several of the major scientific advances discussed. These examples are meant to illustrate the breadth and diversity of the conference, which included many additional presentations that addressed similarly impactful and thought‐provoking topics and underscored the value of bringing together researchers from diverse backgrounds.

## 2 NUCLEAR FUNCTIONS OF CCN PROTEINS IN GENE REGULATION

Experimental data presented by Bernard Perbal, the founder and organizer of the ICCNS‐ARBIOCOM conference meeting, Nice, France, Malini Sen from the CSIR‐Indian Institute of Chemical Biology, India, and Takashi Nishida from the Department of Biochemistry and Molecular Dentistry Okayama University Graduate School of Medicine, Okayama, Japan, collectively support a coherent framework for the functional duality of CCN proteins. Their findings indicate that CCN proteins operate not only as extracellular signaling molecules but also as transcriptional regulators capable of modulating gene expression. As the findings presented in this session have since been reported in multiple publications, only a brief overview of the role of CCN proteins in transcriptional regulation as described by the speakers is provided here. This area is likely to remain a particularly promising avenue for future investigation.

Bernard Perbal reported findings identifying the domains of CCN3 that contribute to its activity as a transcriptional regulator, challenging the prevailing view that CCN proteins act exclusively within the extracellular matrix (ECM). Evidence supporting this concept is threefold.

1. Loss of the signal peptide, which is necessary and sufficient to direct extracellular secretion of CCN proteins, results in targeting of the proteoform to the nucleus. Amino‐truncated CCN3 proteins identified in nephroblastomas and in many tumor cell types are targeted to the nucleus.
2. The yeast two‐hybrid system enabled the identification of a physical interaction between CCN3 and the Rpb7 subunit of RNA polymerase II. Validation using radiolabeled proteins generated by in vitro transcription–translation of candidate clones confirmed that this interaction is not an artifact and can occur independently of the yeast nuclear context.
3. The C‐terminal domain mediates the growth‐inhibitory effects of CCN3. At the molecular level, in vitro transactivation assays indicate that this domain suppresses transcriptional activation driven by the VWC domain within CCN3. The balance between the activities of these two domains is disrupted in nuclear CCN3 proteoforms lacking the amino‐terminal domains. Furthermore, the C‐terminal domain has been shown by others to bind a specific sequence within the PAI‐2 (plasminogen activator inhibitor) promoter, supporting the notion that CCN3 can function as a transcriptional regulator.

In her presentation, Malini Sen provided a synthesis of the multifunctional properties of CCN6, highlighting its roles in both the nucleus and the extracellular matrix (ECM). Work from her group, published more than 2 decades after the initial findings on CCN3 by the Perbal group, provides compelling evidence for a similar functional duality in CCN6. In human chondrocytes and across multiple developmental stages in zebrafish, CCN6 physically associates with RNA polymerase II and plays a critical role in regulating the transcription of genes encoding components of the mitochondrial electron transport chain.

In his presentation, Takashi Nishida reported that CCN2, whose nuclear localization was first described by the Mason group, interacts with the transcription factor PU.1, encoded by the *SPI1* gene. These studies, performed in murine embryonic NIH3T3 fibroblasts, suggest that CCN2 may function as a transcriptional cofactor with potential relevance to fibrotic processes.

Looking ahead, the range of observations outlined above opens a substantial field of investigation that may advance our understanding of the functional duality of CCN proteins and their role in integrating intracellular and extracellular signaling pathways.

Several lines of investigation may further clarify the role of CCN proteins in transcriptional regulation. First, mapping the interaction domains between CCN3 and RNA polymerase II, particularly via the Rpb7 subunit, could help define its potential role in positioning the transcription preinitiation complex (PIC) at the promoters of yet‐to‐be‐identified target genes. Second, binding of the CCN3 C‐terminal domain to the PAI‐2 promoter may exert repressive effects, either directly or through interactions with additional transcriptional regulators. Detailed analysis of plasminogen system regulation by CCN3 and other CCN family members may yield important insights into its broader biological functions. Third, given their nuclear localization, CCN1 and CCN5 are likely involved in various forms of transcriptional regulation.

## 3 EXTRACELLULAR MATRIX (ECM) IN SKELETAL DEVELOPMENT AND DISEASE

Skeletal disorders encompass a broad range of conditions that affect bone, cartilage, and connective tissues, often leading to impaired development, reduced strength, pain, and loss of function. The extracellular matrix is particularly important because its proteins, such as collagens and other structural components, help determine tissue architecture, mechanical stability, and signaling within the skeletal microenvironment. Several presentations examined the mechanisms that drive these disorders with the objective to single out better diagnostic markers and therapeutic targets.

George Bou‐Gharios, the ICCNS awardee from Department of Musculoskeletal and Aging Sciences, Institute of Life Course and Medical Sciences, University of Liverpool, Liverpool, United Kingdom described in a keynote speech how key molecular pathways implicated in fibrosis, including the roles of TGF‐β, endothelin‐1, connective tissue growth factor (CCN2), and matrix regulators affect lung, skin, kidney, and vascular remodeling. His studies provided a mechanistic framework for understanding tissue scarring, degeneration, and repair characteristic of pathological conditions such as systemic sclerosis, lung and kidney fibrosis, and musculoskeletal diseases. A central theme of his work is how the balance between matrix protein synthesis and degradation by matrix metalloproteinases and their tissue inhibitors determines tissue integrity and disease progression. In his elegant presentation, Dr. Bou‐Gharios described his thoughtful approach to identifying a fibroblast‐specific enhancer of the collagen type I alpha 2 gene (COL1A2), revealing how collagen expression is tightly regulated in a cell‐ and tissue‐specific manner and establishing a model for matrix gene regulation in connective tissues. Dr. Bou‐Gharios has developed sophisticated transgenic reporter mouse models that track collagen I production and other matrix‐regulating cells in vivo. These tools that are now widely used to study fibrosis, scarring, and tissue remodeling and to test potential antifibrotic therapies. Extending his expertise in matrix biology, Dr. Bou‐Gharios has investigated cartilage degeneration and osteoarthritis, identifying regulatory elements within the aggrecan gene and testing strategies to protect cartilage from enzyme‐mediated breakdown. Dr. Bou‐Gharios has also contributed to understanding how matrix regulators and signaling pathways influence bone formation, skeletal integrity, and age‐related degeneration, linking basic matrix biology to clinically relevant musculoskeletal disease. Collectively, Dr. Bou‐Gharios's studies have shaped current concepts of how gene regulation in ECM‐producing cells underpins fibrosis, arthritis, and other connective tissue pathologies. By bridging fundamental molecular insights with translational animal models, his research provides a powerful platform for the development of novel therapies aimed at preserving connective tissue structure and function across multiple organs.

Hsu Myat Paing from Okayama University Graduate School of Medicine, Dentistry and Pharmaceutical Sciences, Okayama, Japan, gave a talk entitled “Combination of low intensity pulsed ultrasound and bmp2 enhances the early osteogenic differentiation commitment of mesenchymal stem cells via increased ccn1 production”. This talk covered mesenchymal stem cells (MSCs) which possess self‐renewal and pluripotency, displaying an inverse relationship between adipogenesis and osteoblastogenesis. Although Low‐Intensity Pulsed Ultrasound (LIPUS) inhibits adipogenesis, it alone cannot induce osteoblastic differentiation. This study examined whether combining low‐intensity pulsed ultrasound (LIPUS) with bone morphogenetic protein 2 (BMP2) enhances early osteogenic commitment in C3H10T1/2 murine MSCs. Results showed that LIPUS plus BMP2 synergistically increased Runx2 and markedly upregulated CCN1, CCN2, and CCN4 gene expression, with significant CCN1 protein elevation. These results suggest that CCN1 mediates the enhanced osteogenic differentiation promoted by combined LIPUS and BMP2 stimulation.

Holly Ingraham from the University of California, San Francisco Mission Bay Campus, San Francisco, CA, USA, presented a lecture presentation entitled “CCN3 ‐ a circulating hormone to preserve the female skeleton during pregnancy and lactation”. Dr. Ingraham's presentation was about hormone‐responsive brain–gut circuits that support metabolic and skeletal homeostasis. Notably, Dr. Ingraham discovered a population of hypothalamic KNDy neurons that selectively express CCN3, an osteoanabolic hormone produced exclusively in females and robustly secreted by the brain during lactation. Dr. Ingraham showed that CCN3 acts directly on bone‐derived skeletal stem cells to expand their pool and enhance osteogenic capacity. Her studies positioned CCN3 as a sex‐specific regulator of bone biology and a promising therapeutic target for improving bone health and advancing women's health.

Thomas Ambrosi from the University of California Davis, Sacramento, CA, USA, gave a talk entitled “Opposing forces in bone biology: CCN3 and CCN5 as regulator of skeletal stem cell activity and aging.” Dr. Ambrosi identified CCN5 as a brain‐derived endocrine factor that preserves bone mass during lactation by stimulating stem cell‐mediated osteogenesis. Conversely, recombinant CCN3 enhances osteogenesis in a sex‐independent manner by directly binding skeletal stem cells and osteoprogenitors, with integrin α6 (CD49f) required for activation. CCN5 expression may impair osteogenesis and promote inflammatory hematopoietic activity. Nanobody‐mediated inhibition of CCN5 restores skeletal regeneration, and increases bone mass in aged mice.

Takako Hattori from Okayama University Graduate School of Medicine, Dentistry and Pharmaceutical Sciences, Okayama, Japan, gave a lecture presentation entitled “CCN3 is a senescence marker of cartilage.” Dr. Hattori showed that CCN3 expression positively correlated with p21 and p53 levels in murine chondrocytes across aging, and was upregulated under oxidative stress or exogenous CCN3 stimulation. Cartilage‐specific CCN3‐overexpressing mice exhibited early joint degeneration and elevated senescence‐associated secretory phenotype (SASP) gene expression. Her single‐cell RNA sequencing showed CCN3 enrichment in CD44‐positive progenitor‐like cells suggesting that CCN3 is a key mediator and potential biomarker of chondrocyte senescence driving osteoarthritic cartilage degeneration.

## 4 VASCULAR HOMEOSTASIS, CELL DIFFERENTIATION, CIRCADIAN RHYTHM, AND VASCULAR DISEASES

Several presentations highlighted the diverse roles of matricellular proteins and inflammatory mediators in coordinating vascular remodeling, metabolic signaling, and cellular plasticity across multiple tissues.

Brahim Chaqour from the University of Pennsylvania, Perelman School of Medicine, Philadelphia, PA, USA, gave a thoughtful lecture–presentation entitled “Role of CCN1 in vascular remodeling and regression.” His presentation centered on process of blood vessel formation, regression, remodeling, and their role in retinal microangiopathies. Dr. Chaqour described how defective pruning and regression of fetal or adult pathological blood vessels (BVs) underlie a broad spectrum of congenital and ischemic macro‐ and microangiopathies, including persistent fetal vasculature syndrome, retinopathy of prematurity, and proliferative diabetic retinopathy. These neovascular disorders are marked by excessive formation of immature, leaky vessels, or the persistence of fetal vasculature that fails to undergo timely involution, ultimately resulting in retinal detachment, hemorrhage, and progressive visual impairment. Physiological vascular remodeling requires the coordinated regulation of angiogenic and matricellular cues, including CCN1, which functions as a critical extracellular modulator of endothelial behavior. His endothelial‐specific loss of CCN1 mouse model showed a disruption of normal vascular homeostasis, leading to aberrant vascular hyperplasia, pericyte depletion, and the emergence of disorganized retinal networks lacking hierarchical architecture. Transcriptomic analyses identified matrix metalloproteinase‐14 (MMP‐14) as a downstream effector of CCN1 signaling. MMP‐14, a membrane‐anchored protease that activates MMP‐2 and MMP‐13, plays a pivotal role in vascular involution and ECM remodeling. Notably, MMP‐14 is predominantly expressed by macrophages, which orchestrate developmental vessel pruning by eliminating superfluous branches to align perfusion with metabolic demand. His studies showed that CCN1‐deficient and MMP‐14‐null mice exhibit persistent hyaloid vasculature and increased retinal vascular density, underscoring the necessity of CCN1‐driven MMP‐14 expression in macrophage‐mediated vessel regression. Dr. Chaqour concluded that CCN1‐MMP‐14 signaling is a fundamental mechanism governing fetal vascular involution and may serve as potential therapeutic target for neovascular and congenital vascular pathologies.

Joon‐Il Jun from the University of Illinois at Chicago, College of Medicine, Chicago, IL, USA, gave a talk entitled “CCN1 matricellular signaling links metabolic reprogramming to cellular dedifferentiation for intestinal regeneration.” Dr. Jun described how CCN1 also orchestrates intestinal regeneration following injury. Using single‐cell RNA sequencing and functional studies, his group identified a biphasic regenerative process in which differentiated intestinal cells first dedifferentiate into fetal‐like metaplastic regenerative stem cells and subsequently redifferentiate into mature epithelial lineages. Mechanistically, CCN1 drives this plasticity by activating YAP signaling and inducing metabolic reprogramming through HK2‐mediated glycolysis and lactate production, which promotes histone lactylation and chromatin remodeling at genes controlling cellular plasticity. These findings illustrate how extracellular matrix‐derived signals integrate metabolic and epigenetic mechanisms to regulate tissue regeneration.

Ralf Weiskirchen from the Institute of Molecular Pathobiochemistry, Experimental Gene Therapy and Clinical Chemistry (IFMPEGKC), RWTH University Hospital Aachen, Aachen, Germany, gave a review lecture entitled “Lipocalin 2 (Lcn2), a central mediator of hepatic inflammation and disease.” Dr. Weiskirchen focused on Lipocalin‐2 (LCN2), an iron‐binding siderophore‐sequestering protein that functions as a central mediator of hepatic inflammation and metabolic regulation. His group showed that LCN2 acts as an intrinsic injury signal that promotes inflammatory responses through NF‐κB/STAT signaling pathways. In addition, LCN2 directly regulates the expression of the lipid‐droplet protein Perilipin‐5, thereby influencing lipid‐droplet turnover and mitochondrial β‐oxidation. Loss of LCN2 leads to hepatocyte apoptosis, endoplasmic reticulum stress, and alterations in gut microbiota composition, positioning the LCN2‐Perilipin‐5 axis as an important node linking immune signaling, iron metabolism, and lipid homeostasis in liver disease.

Zhiyong Lin from Emory University School of Medicine, Cardiology Division, Atlanta, GA, USA, presented his work entitled” Safeguarding the vasculature: the matricellular protein CCN2 in vascular homeostasis and protection.” Dr. Lin presented evidence that CCN2 plays a protective role in vascular smooth muscle cell homeostasis and vascular disease. Using smooth muscle cell‐specific CCN2 deletion models, his group demonstrated that CCN2 deficiency promotes the formation of abdominal aortic aneurysms and accelerates atherosclerosis. Mechanistically, loss of CCN2 disrupts TGF‐β signaling and drives pathological phenotypic switching of vascular smooth muscle cells, including their transdifferentiation into macrophage‐like cells. Single‐cell RNA sequencing further revealed increased endoplasmic reticulum stress, endocytosis, and lipid accumulation in CCN2‐deficient cells, underscoring the importance of CCN2 in maintaining vascular wall integrity.

## 5 CELL GROWTH REGULATION AND TUMORIGENESIS

The meeting also highlighted emerging insights into how matricellular proteins and associated signaling pathways regulate tumor progression and cancer cell plasticity.

Anie Philip from McGill University, Montreal, Canada, gave a talk entitled “CD109: the membrane signaling hub that tames fibrosis but fuels cancer.” Her presented work related to CD109, a glycosylphosphatidylinositol‐anchored membrane protein that functions as a molecular signaling hub with context‐dependent roles in fibrosis and cancer. CD109 acts as a coreceptor that inhibits TGF‐β signaling by promoting receptor degradation and turnover, thereby suppressing fibrotic responses. However, recent findings indicate that CD109 can also promote tumor progression in squamous cell carcinoma by enhancing oncogenic signaling pathways, including epidermal growth factor receptor and interleukin‐6 receptor signaling. These observations illustrate how modulation of receptor cross‐talk by CD109 can differentially regulate tissue repair and tumorigenesis.

Celina Kleer from the University of Michigan, Ann Arbor, MI, USA, gave a presentation entitled “Unraveling mechanisms of differentiation of aggressive metaplastic breast cancer.” Dr. Kleer discussed mechanisms underlying the aggressive phenotype of metaplastic breast carcinoma, a rare subtype of triple‐negative breast cancer characterized by high metastatic potential, chemoresistance, and poor response to immunotherapy. Her group identified CCN6 as a tumor suppressor whose loss promotes tumor development in a mammary epithelial cell‐specific knockout mouse model that recapitulates key features of human disease. Mechanistic studies revealed that CCN6 antagonizes pro‐invasive Wnt/β‐catenin/TCF/EZH2 signaling. In CCN6‐deficient tumors, EZH2 overexpression drives sarcomatoid differentiation and promotes chondrogenic and osteogenic gene expression through epigenetic reprogramming, whereas blockade of Wnt/β‐catenin signaling reduces EZH2 levels and activates multiple bivalent gene targets involved in development and differentiation. These findings identify EZH2‐dependent epigenetic regulation as a key driver of differentiation and tumor progression in metaplastic breast cancer.

Together, these presentations emphasized how dysregulation of extracellular matrix signaling, receptor crosstalk, and epigenetic control mechanisms contribute to tumor initiation, cellular plasticity, and malignant progression, highlighting potential therapeutic targets for aggressive cancers.

## 6 CONCLUSIONS

Over the past quarter of a century, the International CCN Society has organized workshops and conferences aimed at advancing our understanding of the biology and pathophysiology of the cellular communication network family of proteins. ARBIOCOM's vision/initiative, now entering its fifth year, was a significant expansion of the ICCNS scope, moving beyond the CCN proteins to include all topics on signaling networks involved in diverse biological systems. It was refreshing to see that this 13th international workshop on the CCN family of genes and inaugural ARBIOCOM conference concluded with resounding success, reflecting exceptional organization and meticulous planning from start to finish. Each scientific session featured outstanding presentations (some of which are summarized above and others are appended to this report) that stimulated scientific exchanges and thoughtful debates. The discussions were not only intellectually rich but also remarkably collegial, fostering a collaborative environment that encouraged the open sharing of ideas. Particularly inspiring was the eagerness of all attendees to take advantage and use state‐of‐the art methodologies and technologies that were not available 10 or 20 years ago to investigate and better understand the function of their gene, protein, pathways or biological process of interest. The ARBIOCOM initiative offered a unique opportunity of cross‐pollination of ideas for addressing many fundamental scientific questions and tackling pressing clinical issues, pushing the boundaries of what is possible in the study of intra‐ and extracellular communication. Presentations from new and established investigators alike were of outstanding quality, showcasing cutting‐edge research and sparking lively yet collegial discussions. A particular highlight was the interaction between senior scientists and young investigators. The latter left the meeting thrilled, motivated, and enlightened by the generosity and wisdom of their mentors.

**FIGURE 4:**
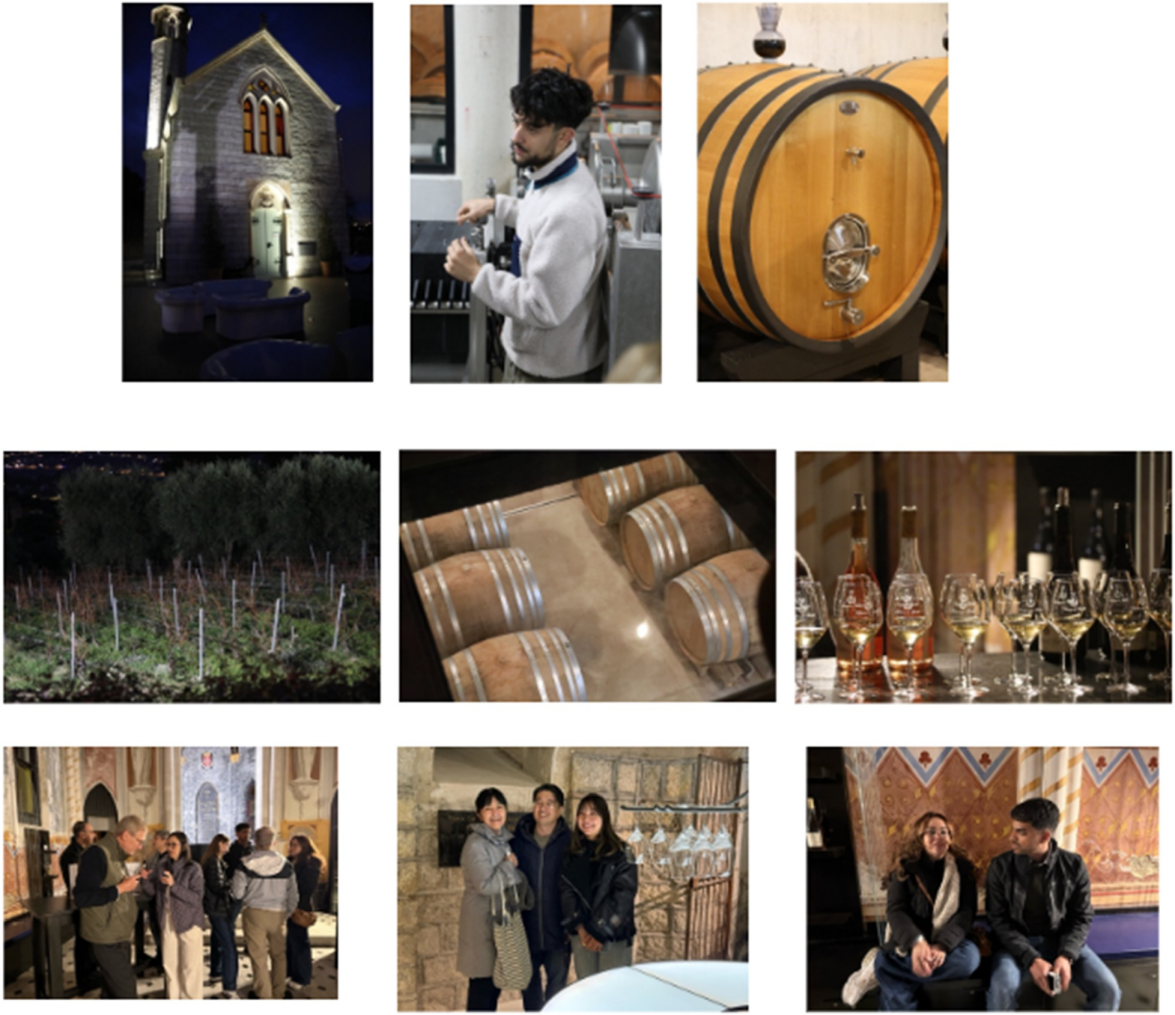
After an intensive and highly productive scientific program, participants enjoyed a visit to Château de Bellet, offering a unique opportunity for prestigious wine tasting and an evening tour of the historic winery. The vineyard is located on the hills overlooking the western side of the city of Nice, at elevations ranging from 200 to 400 m above sea level. On the same evening, the editors of JCCS were invited to a renowned traditional trattoria in Nice for a typical Mediterranean dinner.

**FIGURE 5:**
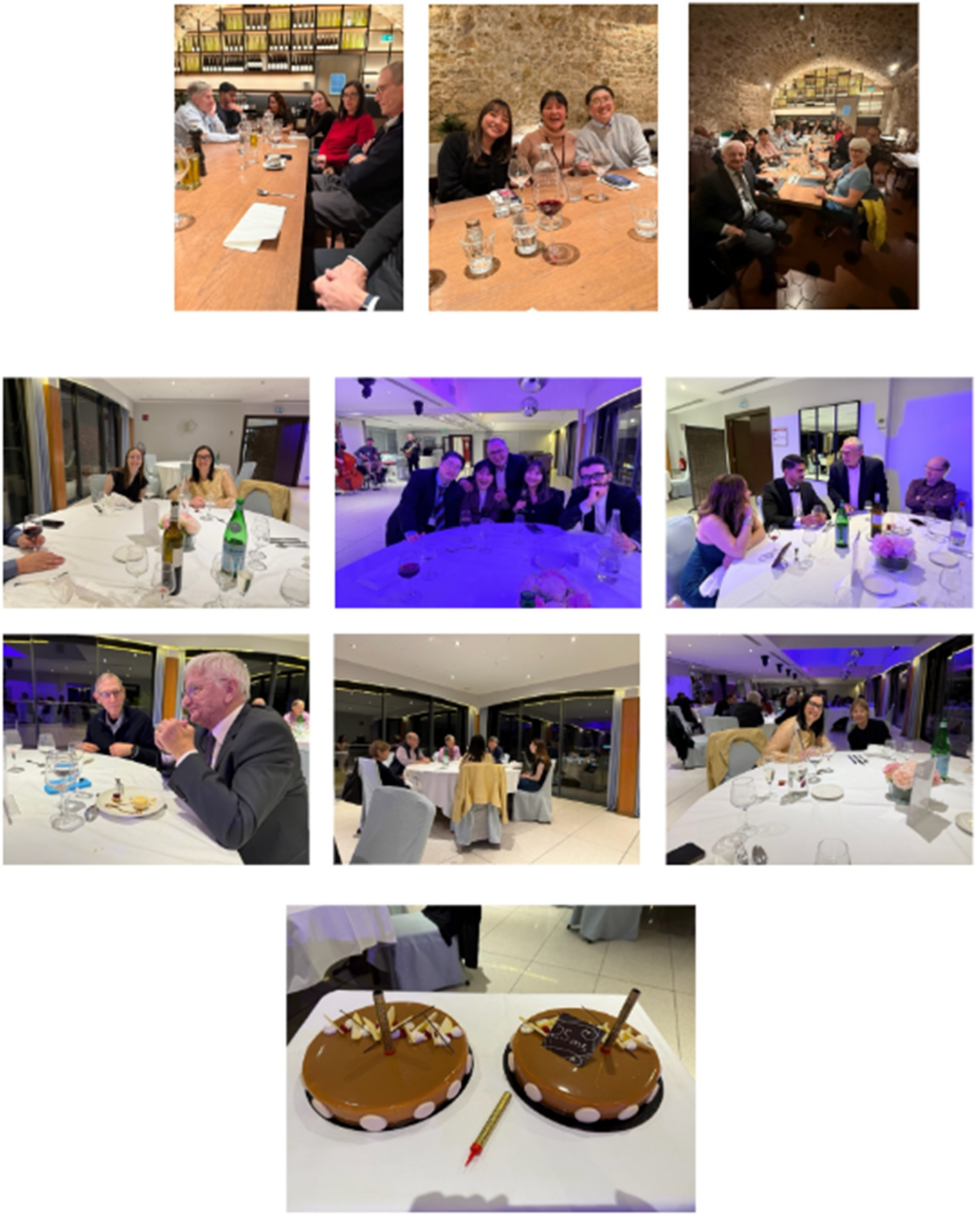
The meeting concluded with an elegant gala dinner accompanied by live musical entertainment, celebrating both the success of the conference and the 25th anniversary of the founding of the ICCNS in 2000.

Over several days, the participants shared groundbreaking research, explored new technologies, and debated fresh ideas in an atmosphere of genuine collegiality and collaboration. Complementing the scientific program attendees also enjoyed enriching social activities including cultural excursions, vin du terroir tasting (Figure 4), lively musical evenings, and fine dinings (Figure 5) that strengthened professional connections and promoted a sense of community among them. The conference concluded with renewed enthusiasm and a collective commitment to advancing discovery in the evolving research field of cellular communication and signaling. Overall, the meeting–conference was a memorable and productive gathering that rekindled enthusiasm for discovery and collaboration among all participants.

## 7 LIST OF ABSTRACTS OF TALKS PRESENTED AT THE 13TH INTERNATIONAL WORKSHOP ON THE CCN FAMILY OF GENES AND INAUGURAL ARBIOCOM CONFERENCE

### 7.1 Opposing forces in bone biology: CCN3 and CCN5 as regulator of skeletal stem cell activity and aging

Thomas Ambrosi

Department of Orthopedic Surgery, University of California Davis, Sacramento, CA, USA

The increase in the prevalence of musculoskeletal conditions, such as osteoarthritis and osteoporosis, imposes a significant clinical and economic burden. The dearth of efficacious preventative and regenerative approaches necessitates innovative strategies. Skeletal stem cells (SSCs), the source of all mature bone, cartilage, and hematopoiesis‐supporting cells, are a promising target to treat bone aging and disease. Intriguingly, CCN proteins have emerged as major players in the regulation of SSC function across life stages. We recently identified CCN5 as a new brain‐derived circulating hormone that sustains bone mass during lactation of female mice by stimulating stem cell‐based bone formation to counteract bone loss. Recombinant CCN3 promotes osteogenesis in vitro and in vivo independent of sex making it an attractive target for an urgently needed osteoanabolic drug. Studies with primary mouse and human cells demonstrated that labeled CCN3 directly and quite selectively binds to SSCs and committed osteoprogenitors. Although a potential receptor remains elusive, interaction with integrin alpha‐6 (CD49f) is crucial for CCN3‐mediated SSC activation. In contrast, CCN5 is one of the most upregulated genes in aged, dysfunctional SSCs. Our studies could show that CCN5 impairs skeletogenesis by SSCs, drives bone loss and induces pro‐inflammatory output from the hematopoietic compartment. Excitingly, nanobody therapy against CCN5 has proven efficient to reactivate aged SSCs, enhance regenerative capacity and improve bone mass in 2‐year‐old mice. Ongoing epigenetic studies explore distinct transcription factor binding sites differentially accessible in aged SSC that will provide greater clarity on CCN5 regulation. Altogether, our work identifies two CCN proteins with opposing roles in bone stem cell biology that could be leveraged for translational approaches against osteoporosis and beyond.

### 7.2 Targeting efferocytosis and phosphatidylserine externalization in the tumor microenvironment for therapeutic applications

Raymond B. Birge *

Rutgers New Jersey Medical School, Department of Microbiology, Biochemistry, and Molecular Genetics, Center for Cell Signaling, Newark, New Jersey, USA (*speaker).

The physiological fate of cells that die by apoptosis is their rapid recognition and clearance by both nonprofessional and professional phagocytes (efferocytosis). Apoptotic cells, by externalizing phosphatidylserine (PS) from the internal to the external surface of the plasma membrane, act as negative regulators of immune responses, promoting immune‐suppression and the resolution of inflammation under physiological conditions to maintain tissue tolerance. In contrast to the homeostatic function of efferocytosis under physiological conditions, tumors in particular exist in a chronic dynamic balance of proliferation, metabolic stress, and apoptosis that magnify the postmortem immunosuppressive effects of apoptotic cells in the tumor microenvironment (TME). Persistent dying cells, as well as stressed viable cells in the TME provide conditions for chronic and constitutive PS exposure that suppresses host tumor immunity by engaging a series of PS receptors called TIM (T cell, Immunoglobulin, and Mucin) and TAM (Tyro3, Axl, and Mertk) receptors. Here we will discuss the role of externalized PS in the tumor microenvironment, the biology of PS scramblases TMEM16F and Xkr8, the mechanisms by which PS is recognized by Mertk, and the function of PS and PS‐receptors in immune evasion. We will describe strategies to target the PS/PSR axis in cancer, including PS targeting mAbs Bavituximab and 11.31 and antiMertk mAb, with therapeutic goals to improve host antitumor immune responses. Finally, we will discuss a novel class of PS‐targeting type I and type III IFN fusion proteins that target immunogenicity to the tumor microenvironment. Our studies support a molecular rationale that PS targeting therapeutics can have utility in cancer immunotherapy.

### 7.3 From gene transcription and cell‐specific regulation to animal models of connective tissue disease

George Bou‐Gharios

Department of Musculoskeletal and Aging Sciences, Institute of Life Course and Medical Sciences, University of Liverpool, Liverpool, UK

The extracellular matrix (ECM) forms the structural framework of connective tissues, and many ECM genes display cell‐type‐specific expression patterns. We have leveraged this property by identifying enhancer elements within gene sequences that drive cell‐ or tissue‐specific transcription, enabling the development of animal models that trace and manipulate cells regulating particular ECM genes.

In our early work, we relied on DNase I hypersensitivity mapping to identify potential enhancer regions. Today, our approach is guided by the ENCODE encyclopedia, which greatly improves our accuracy. We focus on three hallmark histone modifications associated with active enhancers—mono‐methylation of histone H3 lysine 4 (H3K4me1), acetylation of H3 lysine 27 (H3K27ac), and tri‐methylation of H3 lysine 4 (H3K4me3) combined with phylogenetic conservation to pinpoint multiple cis‐regulatory elements. Using this approach, we identified a fibroblast‐specific enhancer in the *Col1a2* gene, which allowed us to visualize and characterize cells expressing collagen type I. Interestingly, these cells were not limited to fibroblasts, revealing new insights into the mechanisms of fibrogenesis and uncovering signaling pathways that influence this process in lung, skin, and kidney. In the context of osteoarthritis, where excessive degradation of ECM proteins leads to cartilage breakdown, we identified several enhancers within the aggrecan (ACAN) gene. By harnessing these enhancers, we expressed regulatory genes such as CCN2, helping to elucidate their role in maintaining articular cartilage integrity. We also utilized *aggrecan*‐derived enhancers to deliver tissue inhibitors of metalloproteinases (TIMPs), which block matrix metalloproteinases (MMPs), thereby mitigating cartilage degradation.

Given that MMP13 plays a central role in ECM destruction in osteoarthritis, we have also focused on dissecting its regulatory mechanisms—an ongoing line of investigation. Together, these studies have established a versatile experimental framework that enables the identification, ablation, or targeted treatment of pathogenic cell populations involved in fibrosis and osteoarthritis, providing new avenues for therapeutic intervention.

### 7.4 Role of CCN1 in vascular remodeling and regression

Sangmi Lee and Brahim Chaqour

Department of Molecular Ophthalmology, Perelman School of Medicine, University of Pennsylvania, Philadelphia, PA, USA

Defective pruning of fetal or adult abnormal blood vessels (BVs) is implicated in diverse congenital and ischemic macro‐ and microangiopathies, including persistent fetal vasculature syndrome, retinopathy of prematurity, and proliferative diabetic retinopathy. These neovascular diseases are characterized by excessively formed leaky BVs or persistence of fetal BVs that fail to regress, leading to retinal detachment, hemorrhage, and visual impairment. Physiological remodeling of the vasculature requires tight regulation by angiogenic molecules, with CCN1 as a key matricellular protein facilitating these processes. Loss of CCN1 function in endothelial cells, one of its major cellular sources, results in pathologic vascular hyperplasia, pericyte loss, and the formation of disorganized retinal networks devoid of normal vessel hierarchy. Transcriptomic profiling identified MMP‐14 as one of the downstream target of CCN1 signaling. MMP‐14 is a membrane‐bound protease that activates MMP‐2 and MMP‐13 and promotes vascular involution suggesting that CCN1‐driven MMP‐14 is essential for proper vessel remodeling.

MMP14 is largely expressed by macrophages, which are known to guide regression and remodeling in the developing mouse vasculature by eliminating excess branches to match perfusion to metabolic demand. Macrophages are also critical for involution of fetal hyaloid vessels (HVs), an exquisitely precise developmental event in which fetal vessel segments are selectively eliminated from an otherwise healthy tissue. Constitutive CCN1 deletion in mice was associated with persistent HVs and impaired regression, whereas wild‐type animals showed complete HV involution by postnatal day 16. Similarly, MMP‐14 knockouts showed increased postnatal retention of HVs and elevated retinal vascular density, consistent with the role of the membrane‐associated MMP‐14 in mediating apoptosis and developmental vessel pruning via macrophage‐vascular cell interactions. Collectively, CCN1‐induced MMP‐14 expression is a critical mechanism for vessel regression and fetal vascular involution, revealing potential therapeutic targets for neovascular and congenital vascular disorders.

### 7.5 Signaling pathways involved in the aging and rejuvenation of human skin

Gary J. Fisher

Department of Dermatology, University of Michigan Medical School, Ann Arbor, MI, USA

Aging is a complex biological process that impacts the functions of all organ and cell systems. It is mainly viewed as a cell‐centered process, involving harmful metabolic, epigenomic, genomic, and inflammatory cellular activities that reduce resilience to stress, leading to frailty. Although this perspective is well supported, it overlooks the fact that all organs contain and interact with an insoluble, protein‐rich extracellular matrix that provides structure, mechanical properties, functions, and the substrate for cellular communication. Connective tissues, such as fascia, tendons, ligaments, and bone, are essential for mobility and are primarily composed of extracellular matrix. Decreased mobility, a condition commonly observed in the elderly, is largely attributed to impaired function of the extracellular matrix‐rich connective tissues.

Collagens, which form the core of the extracellular matrix, make up about 30% of total protein in humans. Type I collagen is the most abundant protein in the human body. With aging, deleterious changes occur in the structure, organization, and composition of the extracellular matrix. These changes are recognized as key, cell‐autonomous hallmarks of aging.

Most of the skin is made up of a collagen‐rich dermal extracellular matrix. Both aging and environmental exposure (the exposome) gradually damage the structure and function of this matrix. Without mechanisms for natural repair, this deterioration accumulates, leading to thinning, fragility, signs of aging such as wrinkles and uneven tone, and an increased risk of diseases, including cancer.

Ultraviolet irradiation from the sun is the primary extrinsic factor that causes skin damage. This damage, referred to as photoaging, is superimposed on natural, intrinsic, chronological aging. Both intrinsic and extrinsic aging are active biological processes driven by numerous signaling mechanisms, including photochemistry, oxidative stress, protein kinases, transcriptional, and matricellular pathways. Age‐related deleterious alterations of the dermal extracellular matrix are inevitable. However, therapeutic interventions that induce cutaneous wound healing, acting through signaling pathways distinct from those that cause aging, can induce the de novo production of extracellular matrix, helping to counteract age‐related deterioration and improve skin health.

Skin aging is visible. It provides an accessible window into the biology of aging, enabling the study of key mechanisms of communication between cells and their extracellular environment, and highlighting the vital role of the extracellular matrix in the process of aging.

### 7.6 CCN3 is a senescence marker of cartilage

Takako Hattori, Janvier Habumugisha, Ryuichiro Okuda, Kazuki Hirose, Miho Kuwahara, Hiroshi Kamioka, and Satoshi Kubota

Okayama University Graduate School of Medicine, Dentistry and Pharmaceutical Sciences, Okayama, Japan

Aging is a major risk factor for osteoarthritis (OA), and degenerative changes in articular cartilage are highly associated with chondrocyte senescence. Here, we focused on the role of cellular communication network factor 3 (CCN3), which has multiple physiological functions during cartilage development, in age‐related changes of cartilage.

The mRNA levels of *Ccn3* and cell cycle arresting genes in primary cultured murine chondrocytes from embryonic day 18.5–37 months of age were examined, and *Ccn3* as well as *p21* and *p53* expressions showed a positive correlation with age. Artificial senescence induced by H_2_O_2_ in RCS cells increased *Ccn3*, *p21*, and *p53* expressions. Besides, exogenous CCN3 protein increased the expression of *p21* and *p53* in RCS cells.

Cartilage‐specific *Ccn3*‐overexpressing mice showed degradative changes in joints even in young animals. Enhanced expression of senescence‐associated secretory phenotype (SASP) factor genes was also found in the rib chondrocytes in those mice.

In human OA femoral head cartilage, CCN3 expression was significantly higher than the control, but no differences were observed between weight‐bearing and nonweight‐bearing parts. CCN3 expression showed a positive correlation with the Mankin score.

To see an impact of CCN3 deletion on OA progression, a mouse model of destabilization of the medial meniscus (DMM) was produced. CCN3 knockout (KO) mice harbored DMM surgical displayed less cartilage degradation and reduced proteoglycan loss as well as suppressed production of matrix‐degrading enzymes such as MMP‐13 and ADAMTS‐5, resulting in protection against cartilage damage. Increased expression of KI‐67 in KO‐DMM cartilage suggests enhanced chondrocyte proliferation.

By analyzing single‐cell RNA sequencing (scRNA‐seq) data of cartilage from a model of osteoarthritis, enriched CCN3 was found in mesenchymal stem cell populations in which CD44 coexpressed, suggesting a link between CCN3 and cartilage progenitor‐like phenotypes. In fact, WT OA cartilage showed more CCN3‐CD44 double‐positive cells.

In vitro experiments showed that stimulation of RCS cells with IL‐1β markedly upregulated *Ccn3*, *Mmp13*, and *Adamts5* expressions. However, *Ccn3* knockdown significantly suppressed these inflammatory and catabolic responses, indicating that *Ccn3* is required for the upregulation of these mediators under pro‐inflammatory conditions.

Overall, those data demonstrate that CCN3 can be a new marker of chondrocyte senescence, and CCN3 induction promotes chondrocyte senescence, leading to degenerative changes in articular cartilage through p53 and p21. CCN3 expression was associated with the severity of OA. Finally, *Ccn3* deficiency mitigates OA progression by inhibiting catabolic mediators and promoting chondrocyte proliferation.

### 7.7 CCN3—A circulating hormone to preserve the female skeleton during pregnancy and lactation

Holly A. Ingraham^1^, Zsofia Torok^1^, Deepanshu Soota^1^, Kun Chen^2^, and Thomas Ambrosi^2^

^1^University of California, San Francisco Mission Bay Campus, San Francisco, CA, USA

^2^ University of California, Davis, Medical School, Sacramento, CA, USA

Biological sex is one of nature's most fundamental variables. Leveraging sex differences in mice as our model system, we have uncovered how hormone‐responsive nodes in the brain and gut adapt to support metabolic and skeletal health. Relevant to this conference, we recently identified a group of hypothalamic neurons that produce CCN3, an osteoanabolic hormone produced specifically in female KNDy neurons and secreted by the brain during lactation. Ablating *Ccn3* expression in the female brain lowers circulating levels of CCN3, degrades the maternal skeleton, and leads to premature death of newly born offspring. In vitro and in vivo evidence suggests that CCN3 acts directly on bone‐derived skeletal stem cells to expand their numbers and boost their bone‐forming capacity. Before birth, CCN3 is produced by endocrine cells in the placenta, prompting us to assess the functional role of placental CCN3 in maintaining the maternal and fetal skeletons. Ongoing efforts also seek to identify the cellular targets unique to CCN3 and how *Ccn3* transcripts are dramatically upregulated in KNDy neurons during lactation. We propose CCN3 as a highly relevant target for rejuvenating bone quality and function and improving women's health.

### 7.8 CCN1 matricellular signaling links metabolic reprogramming to cellular dedifferentiation for intestinal regeneration

Naiyuan Cheng, Seungwon Shin, and Joon‐Il Jun

Department of Biochemistry and Molecular Genetics, College of Medicine,

University of Illinois at Chicago, Chicago, IL, USA

Tissue regeneration following crypt injury or loss of intestinal stem cells (ISCs) requires transient fetal reprogramming, yet the molecular mechanisms enabling such plasticity remain poorly understood. Here, we identify an uncharacterized biphasic intestinal regenerative trajectory in which differentiated cells first reprogram into a fetal‐like stem cell state, which we termed metaplastic regenerative stem cells (mrSCs), and later redifferentiate into secretory and absorptive lineages. Single‐cell RNA sequencing and functional assays revealed that a matricellular protein CCN1 is a key determinant of mrSCs reprogramming through chromatin remodeling, specifically via histone H3K9 and H3K18 lactylation. Mechanistically, CCN1 activated YAP and, induced HK2‐driven glycolysis and lactate production, promoting histone lactylation. CUT&Tag profiling uncovered that lactylated histones were enriched at promoters of plasticity regulators, including KLF4, AP‐1 members, and their target genes, suggesting metabolite‐driven chromatin remodeling underlies dedifferentiation. Although lactate treatment to irradiated Ccn1‐deficient mice restored fetal gene expression, it failed to fully recapitulate the mrSC transcriptomic program, leading to impaired redifferentiation and dysplastic‐like structures. Notably, combined treatment with lactate and soluble JAG1, a Notch ligand, partially rescued these phenotypes. Together, our findings delineate a biphasic model of regeneration in which CCN1 orchestrates both dedifferentiation and redifferentiation via extracellular matrix signaling, metabolism, and epigenetic remodeling.

### 7.9 A neutrophil‐associated proteomic signature in tonsillar T‐cells might drive inflammation in PFAPA syndrome

Feras Kharrat^1^*, Nour Balasan^1^, Valentina Golino^2^, Pietro Campiglia^2^, Erica Valencic^1^, Adamo Pio d’Adamo^1,3^, Alberto Tommasini^1,3^, Egidio Barbi^1,3^, Domenico Leonardo Grasso^1^, Blendi Ura^1^

^1^Institute for Maternal and Child Health, IRCCS Burlo Garofolo, Trieste, Italy.

^2^Department of Pharmacy, University of Salerno, Salerno, Italy

^3^Department of Medicine, Surgery and Health Sciences, University of Trieste, Trieste, Italy.

Periodic Fever, Aphthous Stomatitis, Pharyngitis, and Adenitis (PFAPA) syndrome is a pediatric autoinflammatory disorder with recurrent, sterile oropharyngeal inflammation. Although tonsils are the primary inflammation site, the molecular drivers within key immune cells are poorly defined. T‐cells (CD3+) are crucial immune orchestrators, yet their precise role in the hyper‐inflammatory state of PFAPA is unclear. This study aimed to define the proteomic landscape of tonsillar T‐cells to uncover pathways driving the disease.

Six patients with PFAPA and six patients with OSAS were included in the study. CD3+ cells were isolated from the tonsillar tissue of the patients using magnetic microbeads. Proteins were extracted from the isolated cells. Protein extracts were digested and analyzed by nano‐liquid chromatography–mass spectrometry on an Orbitrap Lumos using both Data‐Dependent (DDA) and Data‐Independent Acquisition (DIA) modes. Raw DDA and DIA data were processed with Proteome Discoverer 3.1 and Spectronaut 19, respectively, at a 1% FDR. Pathway and functional bioinformatics analyses were conducted using Reactome, SRplot, and Cytoscape.

Proteomic analysis of highly purified tonsillar CD3+ T‐cells revealed a distinct protein signature in PFAPA patients compared to controls with Obstructive Sleep Apnea Syndrome (OSAS). Reactome pathway analysis identified Neutrophil Degranulation (R‐HSA‐6798695) as the most significantly enriched pathway in PFAPA T‐cells, with profound statistical significance (*p* = 2.8e‐5, FDR = 4.5e‐4). A total of 17 proteins from this pathway, including key granule components like S100A8/A9, MPO (Myeloperoxidase), and ELANE (Neutrophil Elastase), were differentially abundant in the T‐cell population from PFAPA patients.

The enrichment of a “Neutrophil Degranulation” pathway in PFAPA T‐cells is a paradoxical finding. It suggests these T‐cells are uniquely programmed to express potent pro‐inflammatory proteins typically associated with neutrophils. This T‐cell intrinsic “neutrophil‐mimicry” could be a key mechanism driving the intense, sterile inflammation seen in PFAPA flares. Our findings uncover a novel, hybrid inflammatory phenotype in PFAPA T‐cells, representing a potential pathogenic hub and a promising new avenue for targeted therapeutic strategies.

### 7.10 Unravelling mechanisms of differentiation of aggressive metaplastic breast cancer

Celina G. Kleer

Department of Pathology, University of Michigan, Ann Arbor, MI, USA

Despite considerable progress in breast cancer treatment, specific subtypes of triple negative breast cancers (TNBC) have proven resistant to conventional and immune therapies with 5‐year survival rates still around 50%. Among the most aggressive of these is metaplastic breast carcinoma (mBrCA), whose features include increased metastasis and chemoresistance, a unique histopathology with spindle or sarcomatoid (chondroid/osseous) components, and low immune cell infiltration with poor responses to immunotherapy. However, the mechanisms underlying these hyperaggressive tumor phenotypes remain unexplored. Our lab has discovered that CCN6 is a tumor suppressor for mBrCA. We generated a mammary epithelial cell‐specific Ccn6 knockout mouse model (MMTV‐Cre; Ccn6^fl/fl^, Ccn6‐KO), which developed mammary tumors similar to human spindle mBrCAs at pathological and proteomic levels. Mechanistically, we found that secreted CCN6 protein antagonizes the pro‐invasive effects of Wnt/b‐catenin/TCF/EZH2 signaling. In this study, we tested the *hypothesis that EZH2 overexpression drives sarcomatoid differentiation in CCN6‐KO mBrCA through epigenetic reprogramming*.

Ccn6‐KO cells derived from MMTV‐Cre; Ccn6^fl/fl^ tumors. Chondrogenic and osteogenic differentiation assays using Alcian Blue or Alizarin red stains were performed in Ccn6‐KO cells with adenovirus‐mediated EZH2 overexpression, lentivirus‐mediated EZH2 knockdown (KD), or controls. qRT‐PCR measured gene expression. Ccn6‐KO cells transduced with dominant negative (dn) TCF4 or control underwent ChIP‐seq using H3K27me3 and H3K4me3 antibodies. Ccn6‐KO EZH2 overexpressing cells or controls were injected into the tibia of FVB mice. We performed Image Tissue CyTOF of 27 human mBrCA tumors. We found that EZH2 overexpression significantly enhances chondrogenic and osteogenic differentiation of Ccn6‐KO cells and upregulates differentiation genes. Sh‐EZH2 KD significantly reduced chondrogenic and osteogenic differentiation abilities and gene expression in Ccn6‐KO cells, which was rescued by EZH2 overexpression. Blockade of Wnt/b‐catenin using dnTCF4 results in downregulation of EZH2 and activation of 19 bivalent gene targets with functions in development and differentiation including ACE, NPR3, DACTA2, and STC1, validated by qRT‐PCR in cells cultured in osteogenic and chondrogenic conditions. In vivo, EZH2 overexpression provides growth advantage to Ccn6‐KO mBrCA cells in the bone microenvironment of the tibia, compared to controls. Human mBrCAs with chondroid differentiation have higher percentage of EZH2+ tumor cells compared to spindle mBrCAs.

EZH2 regulates Ccn6‐KO mBrCA differentiation by inducing a phenotypic switch from spindle to sarcomatoid morphology. We identified novel EZH2‐regulated bivalent loci that may function as epigenetic modulators of differentiation.

### 7.11 Safeguarding the vasculature: The matricellular protein CCN2 IN vascular homeostasis and protection

Zhiyong Lin

Cardiology Division, Emory University School of Medicine, Atlanta, GA, USA

Vascular smooth muscle cell (SMC) phenotypic switching plays a pivotal role in the pathogenesis of aortic diseases, including aortic aneurysm and atherosclerosis. Cellular communication network factor 2 (CCN2), a matricellular protein often upregulated in human vascular pathologies, exerts diverse and context‐dependent biological effects. However, its specific function in SMCs within aortic disease has remained unclear. Using SMC‐restricted CCN2 deletion models, we demonstrate that CCN2 deficiency predisposes mice to both abdominal aortic aneurysm (AAA) and atherosclerosis. In Angiotensin II–infused hypercholesterolemic mice, loss of SMC‐CCN2 promotes AAA formation in the infrarenal aorta, mirroring human disease. Mechanistically, CCN2 deficiency reprograms SMCs by disrupting TGF‐β signaling and altering Kruppel‐like factor 4 and contractile marker expression, driving pathological phenotypic switching. In atherosclerosis, CCN2 deletion similarly accelerates plaque formation, as evidenced by lipid‐rich lesions and increased transdifferentiation of SMCs into macrophage‐like cells. Single‐cell RNA sequencing and in vitro studies reveal that CCN2 loss increases endoplasmic reticulum stress, endocytosis, and lipid accumulation in both mouse and human SMCs. Collectively, these findings uncover a previously unrecognized protective role of SMC‐derived CCN2 in maintaining vascular wall homeostasis and identify it as a critical regulator of SMC plasticity underlying aortic aneurysm and atherosclerosis.

### 7.12 Cross‐species comparison of CCN gene expression and regulatory networks in chondrogenic cell cultures

Zhangzheng Wang¹, Judit Vágó¹, Szilárd Pliska², Eun‐Jung Jin³, Bernard Perbal⁴, Csaba Matta¹

¹Department of Anatomy, Histology and Embryology, Faculty of Medicine, University of Debrecen, Debrecen, Hungary

²Genomic Medicine and Bioinformatics Core Facility, Department of Biochemistry and Molecular Biology, Faculty of Medicine, University of Debrecen, Debrecen, Hungary

³Department of Biological Sciences, Wonkwang University, Iksan, Jeonbuk, South Korea

⁴International CCN Society, Nice, France

Cartilage defects and osteoarthritis pose significant challenges due to the limited regenerative capacity of articular hyaline cartilage, necessitating robust translational approaches for tissue repair and regeneration. CCN proteins, a family of matricellular regulators, play pivotal and species‐dependent roles in chondrogenesis, yet their expression patterns and regulatory mechanisms in different model organisms remain incompletely defined. Here, we present a comparative transcriptomic analysis of CCN gene expression and interacting networks across three widely used in vitro chondrogenesis systems: chicken and mouse embryonic limb bud micromass cultures, and human mesenchymal stem cells differentiated into chondrocytes. Bulk RNA sequencing was performed across key differentiation timepoints, revealing striking conservation in overall chondrogenic transcriptomic trajectories among the avian and murine models, whereas the human system displayed analogous but distinct secondary clustering. CCN1 and CCN2 were the predominant family members upregulated during late‐stage chondrogenesis in both avian and murine cultures. Notably, CCN6 was absent in chickens but maintained low baseline expression in mammalian systems. Coexpression network analysis demonstrated that CCN proteins serve as network hubs, with associated gene modules enriched for pathways governing extracellular matrix organization, growth factor signaling, and chondrocyte differentiation. Functional enrichment identified coordinated regulation through pathways such as PI3K‐Akt, Hippo, and HIF‐1, with species‐specific variations in CCN protein interactions and downstream targets.

This comparative approach underscores conserved features and species‐specific nuances in CCN‐mediated regulation of chondrogenesis, providing insights into the selection and translational relevance of in vitro models for cartilage biology and regenerative medicine. The findings inform targeted therapeutic strategies for cartilage repair, addressing critical clinical needs in osteoarthritis and related musculoskeletal disorders.

### 7.13 Interaction between nuclear‐translocated cellular communication network factor 2 and PU.1 regulates the expression of fibrosis‐related genes

Xuan Thi Nguyen,^1^ Satoshi Kubota,^1, 2^ Masaharu Takigawa,^2^ Takashi Nishida,^1^*

^1^Department of Biochemistry and Molecular Dentistry, Okayama University Graduate School of Medicine, Dentistry and Pharmaceutical Sciences, Okayama, Japan

^2^Advanced Research Center for Oral and Craniofacial Sciences, Okayama University Faculty of Medicine, Dentistry and Pharmaceutical Sciences, Okayama, Japan

Cellular communication network factor 2 (CCN2) is a matricellular protein with a signal peptide in N‐terminus and a nuclear localization signal‐like peptide around the C‐terminus and is considered to promote fibrosis. However, it is unknown whether or not CCN2 translocates into the nucleus and how nuclear‐translocated CCN2 is involved in the progression of fibrosis. We hypothesized that CCN2 translocates into the nucleus of fibroblasts, and nuclear‐translocated CCN2 interacts with purine‐rich box 1 (PU.1) regulating the differentiation of myofibroblasts, which are key players in fibrosis. We first showed that CCN2 was detectable both in the cytoplasm and nuclei of murine fibroblastic NIH3T3 cells by Western blot analysis of the cytoplasmic and nuclear fractions of cell lysate and immunofluorescence analysis. Additionally, chromatin immunoprecipitation‐PCR and electrophoretic mobility shift assay (EMSA) revealed that recombinant CCN2 protein bound to the regulatory region of *Spi1*, which encodes PU.1. Furthermore, immunoprecipitation‐Western blot analysis showed that CCN2 interacted with PU.1. Finally, to investigate the functions of the complex of CCN2 and PU.1, we performed the forced expression of both *Ccn2* and *Spi1*. The forced expression of both genes significantly promoted fibrosis‐related genes, such as *Col1a1*, *Acta2*, *Ace1*, *Agtr1*, and *Agtr2*. Especially, this complex promoted the production of type I collagen and α‐smooth muscle actin, which are markers of myofibroblasts, and the production of angiotensin II. These findings indicate that CCN2 translocated to the nucleus interacts with PU.1 and that their complex promotes myofibroblast differentiation, suggesting that CCN2 plays an important role in fibrosis via cooperation with PU.1 as a transcription co‐factor.

### 7.14 CCN1 expression is induced by IGF1 and promotes the proliferation of prostate cancer cells through the PI3/AKT signaling pathway

Greisha L. Ortiz‐Hernández^1^, Carmina Patrick^1^, Stefan Hinz^1^, Mark A. LaBarge^1^, Yun R. Li^2^, Susan L. Neuhausen^1^

^1^Department of Population Sciences, Division of Biomarkers of Early Detection and Prevention, Beckman Research Institute of City of Hope, Duarte, CA, USA

^2^Department of Radiation Oncology, City of Hope Comprehensive Cancer Center, Duarte, CA, USA

Prostate cancer (PCa) progression is closely linked to dysregulated growth factor signaling. Cellular Communication Network Factor 1 (CCN1), a matricellular protein with an insulin‐like growth factor‐binding domain, has emerged as a potential modulator of tumor growth. This study investigates the role of CCN1 in IGF1‐mediated PCa cell proliferation and its downstream signaling mechanisms.

Using siRNA‐mediated knockdown in PC3, LNCaP, and 22Rv1 PCa cell lines, we demonstrate that CCN1 is essential for maintaining cell viability, proliferation, migration, and prostasphere formation. CCN1 depletion significantly attenuated these oncogenic behaviors. Mechanistically, CCN1 silencing inhibited PI3K/AKT pathway activation, whereas MAPK signaling remained unaffected. IGF1 treatment upregulated CCN1 expression in a time‐dependent manner, and pharmacological inhibition of PI3K/AKT signaling suppressed both CCN1 induction and cell proliferation.

Furthermore, IGF1 modulated integrin α5 and β1 expression, suggesting a role for CCN1 in cell adhesion and migration. These findings position CCN1 as a critical mediator of IGF1‐driven PCa progression through PI3K/AKT signaling, highlighting its potential as a therapeutic target to limit tumor growth and metastasis.

### 7.15 Combination of low intensity pulsed ultrasound and BMP2 enhances the early osteogenic differentiation commitment of mesenchymal stem cells via increased CCN1 production

Hsu Myat Paing^1,2^, Takashi Nishida^1^, Masaharu Takigawa^3^, Takuo Kuboki,^2^, Satoshi Kubota,^1,3^

^1^Department of Biochemistry and Molecular Dentistry, Okayama University Graduate School of Medicine, Dentistry and Pharmaceutical Sciences, Okayama, Japan.

^2^Department of Oral Rehabilitation and Regenerative Medicine, Okayama University Graduate School of Medicine, Dentistry and Pharmaceutical Sciences, Okayama, Japan.

^3^ARCOCS, Okayama University Faculty of Medicine, Dentistry and Pharmaceutical Sciences, Okayama, Japan.

Mesenchymal stem cells (MSCs), which have the abilities of self‐renewal and pluripotency, are well‐known to exhibit the reciprocal inhibitory relationship between adipogenesis and osteoblastogenesis. Our previous study demonstrated that low‐intensity pulsed ultrasound (LIPUS) effectively suppressed adipogenesis. However, LIPUS alone proved insufficient to induce osteoblastic differentiation by suppressing adipogenesis. Therefore, this study aims to investigate whether or not combining LIPUS with bone morphogenetic protein 2 (BMP2) enhances early osteogenic commitment of MSCs.

Murine MSC cell line, C3H10T1/2, was cultured in osteogenic differentiation medium including 10 μg/mL ascorbic acid and 10 mM β‐glycerophosphate for 10 days. During the final 3 days, the cells were treated with recombinant BMP2 at a concentration of 100 ng/mL along with daily 20‐min LIPUS (3.0 MHz, 60 mW/cm²) treatments. Gene expression of osteoblastic markers and cellular communication network factor (CCN) family members was analyzed by quantitative RT‐PCR. CCN1, CCN2, and CCN4 protein levels were assessed by Western blot analysis.

Similar to our previous data, LIPUS alone had no effect on the gene expressions of osteogenic markers, such as Runx2, Osterix, alkaline phosphatase, and Osteopontin. In contrast, a combination of LIPUS and BMP2 treatment synergistically upregulated *Runx2* expression, although the other marker genes were not affected. Interestingly, combination treatment dramatically upregulated *Ccn1*, *Ccn2*, and *Ccn4* expressions compared to the treatment with LIPUS or BMP2 alone. However, only CCN1 was significantly elevated at the protein level by the combined treatments.

Combined LIPUS and BMP2 treatment significantly increased the gene expression of Runx2, which is related to early osteoblastic differentiation of MSCs. In addition, the combined treatments dramatically promoted CCN1 production. These findings indicate that treatment with both LIPUS and BMP2 plays an important role in early osteogenic differentiation commitment of MSCs via increasing CCN1 production, thus suggesting CCN1's therapeutic relevance in bone regeneration.

### 7.16 Circadian Cryptochrome 1/2 proteins regulate extracellular matrix homeostasis via TGFβ/ALK5 pathway: Implications for connective tissue diseases

Cal S^1^, Sarumi D^1^, Marinkovic M^2^, Peters C^1^, Carter A^1^, Van der Horst GTJ^3^, Kearns V^1^, Poulet B^1^, Bou‐Gharios G^1^, Pekovic‐Vaughan V^1^.

^1^ Department of Musculoskeletal Biology and Aging Science, University of Liverpool, Liverpool, UK

^2^ Department of Orthopedic Surgery, Indiana University School of Medicine, Indianapolis, IN, USA

^3^ Department of Genetics, Erasmus University Medical Center, CA Rotterdam, The Netherlands

Chronic tissue scarring resulting from unresolved tissue repair contributes toward the leading causes of morbidity and mortality worldwide. Recent studies demonstrate that genetic clock disruption in mouse models leads to increased susceptibility of tissues to fibrotic injury. The circadian clock is an endogenous timing system that allows an organism to anticipate and optimally respond to daily changes in the environment over a ∼24h cycle. Human and mammalian cells have an intrinsic biological clock, driven by a conserved set of core clock genes and proteins that form positive/negative feedback loops. Here, we investigated the role of the negative loop components of the clock, Cryptochrome 1/2 proteins, in the regulation of target matrix genes implicated in fibrosis.

The expression of matrix/fibrotic genes was assessed in peripheral tissues from wild type (WT) and fibrotic mice over 24h using qPCR and western blotting. Primary mouse embryonic fibroblasts (MEFs) from WT and *Cry1/2* KO mice (a genetic model of arrhythmic clock) were used to test the rhythmic expression of matrix genes. AFM microscopy was used to assess their stiffness and adhesion properties. The effects of siRNA‐mediated knockdown of *Cry1/2* was tested in a human cell line using qPCR and real‐time bioluminescence imaging. Matrix gene promoter activity was assayed using dual luciferase reporter. Pharmacological or genetic *Cry1/2* rescue was used to assess matrix genes following TGFβ1 stimulation.

Rhythmic expression of several TGFβ target genes implicated in fibrosis is observed in WT mouse tissues. They are regulated, in part, by clock‐responsive elements in their gene promoters. Elevated expression of target matrix genes was observed in *Cry1/2* KO MEFs. TGFβ stimulation led to further induction of matrix genes in *Cry1/2* KO MEFs, which was prevented using canonical TGFβ pathway inhibitors or softer matrix. Cry1/2 KO MEFs display stiffer matrix and stronger adhesion. *Cry1/2* siRNA‐mediated knockdown in human cells exerted profound effects on the TGFβ target genes. Pharmacological stabilization of CRY1/2 proteins reduced TGFβ‐stimulated induction of matrix genes in WT MEFs in vitro and tissues in vivo. Finally, we observed downregulation of *Cry1/2* genes in tissues of fibrotic mouse models and human patient cells from connective tissue diseases.

These results demonstrate that *Cry1/2* represses expression of target matrix genes in a TGFβ‐dependent manner and that fibrotic conditions lead to reduced *Cry1/2* rhythms. Thus, *Cry1/2* may play an important role in the negative feedback regulation of TGFβ pathway during resolution of tissue repair.

### 7.17 CCN3: A two‐voiced part in the fantastic symphony of life’s communication and signaling

Bernard Perbal

International CCN Society, Nice, France

The concept of the CCN family of proteins originates from the structural features shared by its members. It is often implied that, beyond these constitutional considerations, the members of the CCN family also participate in common biological pathways. Yet the more we learn about the functions of each of the six proteins that constitute the CCN family, the more we must consider them as unique entities that share only a few common traits.

Of course, we cannot ignore the unique tetra‐modular organization of these proteins, nor the fact that their modules contain small stretches of conserved sequences found in tens of thousands of other regulatory factors. The temptation to believe that the combination of four functional domains represents a nearly final evolutionary stage is compelling. The assembly, within a single molecule, of four entities whose expression and activities must be coordinated may appear as an exquisite step toward governing a multisignaling axis that prefigures the coordination of the wealth of biological processes underlying all aspects of life.

Therefore, it is important to determine to what extent the biological activity of the whole proteins compares with the sum of the elementary activities contributed by their constitutive domains. A critical question regarding the biological properties and functions of the CCN family has emerged from seminal work identifying CCN3 (aka NOV), and more recently CCN6 (aka WISP3), as potential regulatory transcription factors physically interacting with the RNA polymerase II complex.

In this communication, we will examine some of these aspects from a distance and attempt to broaden our scope and perspective. Until now, we have considered only the CCN sonata in which each of the six instrumentalists engages in specific sets of dialogues with the orchestra. We know this is only part of the puzzle, given the spatiotemporal regulation to which they are subjected. Ultimately, it is essential to establish which instrumentalists need to be present at the same time in the same room.

Rehearsals performed with parts of the orchestra can provide clues about what happens when some of them are absent, or when only a subset can perform. The next stage will consist in identifying a conductor responsible for coordinating all the regulatory factors performing together at various timings in the Fantastic Life Signaling Symphony.

### 7.18 CD109: The membrane signaling hub that tames fibrosis but fuels cancer

Anie Philip

McGill University, Montreal, Canada

CD109 exemplifies how a single membrane protein can function as a molecular signaling hub with diametrically opposing roles in tissue repair and tumor progression. Transforming growth factor‐β (TGF‐β) is a multifunctional growth factor that plays a central role in fibrosis by promoting extracellular matrix production and is among the most potent pro‐fibrotic factors in the body. We previously identified CD109, a glycosylphosphatidylinositol‐anchored membrane protein, as a coreceptor and inhibitor of TGF‐β signaling, and demonstrated that it promotes TGF‐β receptor degradation and turnover, thereby suppressing fibrotic responses in vitro and attenuating fibrosis in vivo. However, emerging evidence from our laboratory and others indicates that CD109 drives tumor progression in multiple cancer cell types. Our recent results show that CD109 promotes tumor progression in squamous cell carcinoma, by enhancing oncogenic pathways such as epidermal growth factor receptor and interleukin‐6 receptor signaling. Recognizing CD109 as a molecular hub that differentially regulates fibrosis and cancer not only reframes its biological significance but also reveals mechanistic vulnerabilities that may be exploited to modulate receptor cross‐talk and reprogram fibrotic or malignant phenotypes.

### 7.19 CCN6: Insights and unexplored frontiers

Malini Sen

CSIR‐Indian Institute of Chemical Biology (Ret.)

CCN6 is one of several proteins that play a critical role in human health and disease. Despite its importance, CCN6 has not been adequately studied, leaving several gaps in our understanding of its structure, function, and mechanism of action. Our aim here is to elucidate the multifaceted functions of CCN6 in relation to its structural organization with particular attention to its association with the musculoskeletal disorder Progressive Pseudo Rheumatoid Dysplasia (PPRD), which arises from mutations in the CCN6 gene. Here we have categorically described the significance of CCN6 in the maintenance of chondrocyte and cartilage health, as well as muscle organization, in relation to PPRD. Our analysis of CCN6 function in relation to its modular structural arrangement highlights its existence both as a secreted protein and in association with cellular organelles such as the mitochondria and the nucleus, suggesting its diverse involvements in cellular signaling at the level of a ligand acting at the cell surface, mitochondrial respiration and transcription in musculoskeletal tissue. In view of several reports linking CCN6 expression level to different forms of cancer, we furthermore, seek to understand if CCN6 function is similar or different in cells and tissues of different origin. Based on our analysis of CCN6 function in relation to the musculoskeletal system, and other reports focusing on CCN6 in relation to cancer we have assembled several important aspects of CCN6 structure–function that demand contemplation for further research in the interest of a comprehensive understanding of CCN6 biology in relation to human health and disease.

### 7.20 Lipocalin 2 (LCN2), a central mediator of hepatic inflammation and disease

Ralf Weiskirchen

Institute of Molecular Pathobiochemistry, Experimental Gene Therapy and Clinical Chemistry (IFMPEGKC), RWTH University Hospital Aachen, Aachen, Germany

Lipocalin 2 (LCN2), also known as neutrophil‐gelatinase–associated lipocalin (NGAL), is a siderophore‐binding transport protein that limits microbial iron acquisition. As a member of the lipocalin family, it has various functions in many organs. However, the way it mediates its cellular effects and its impact on hepatic homeostasis and pathology remains insufficiently defined. In the past, we utilized an *Lcn2* knock out mouse model and a diverse set of complementary liver‐injury models—such as carbon tetrachloride (toxic), lipopolysaccharide (inflammatory), high‐fat diet (steatosis), and bile duct ligation (cholestatic)—to delineate the cellular sources, molecular targets and pathophysiological consequences of LCN2 signaling. A decade ago, we postulated that LCN2 acts as an intrinsic “help me” sensor that, upon injury, develops an activity to recruit inflammatory cells. We have shown that LCN2 in the liver is a central mediator of biosignaling and cellular communication regulated through the NF‐κB/STAT signaling pathway. In more recent data, we discovered that LCN2 directly controls the expression of Perilipin 5, a lipid‐droplet coat protein: LCN2 deficiency suppressed, whereas LCN2 overexpression increased, Perilipin 5 transcription, thereby altering lipid‐droplet turnover and mitochondrial β‐oxidation. Moreover, depletion of *Lcn2* in mice leads to dysbiosis and persistent colonization with segmented filamentous bacteria, resulting in lowered resistance to infection. On the cellular level, the loss of LCN2 provokes hepatocyte apoptosis, unfolded protein, and endoplasmic reticulum stress. We were able to confirm all these findings in human organoid cultures, positioning LCN2 at the nexus of immune‐metabolic crosstalk in the liver, linking iron metabolism to lipid‐droplet dynamics and inflammatory signaling. We highlight the LCN2–Perilipin 5 axis as a promising biomarker‐guided therapeutic target across a spectrum of acute and chronic hepatic diseases. It further serves as a hub of cellular communication during the pathogenesis of cancer.

## AUTHOR CONTRIBUTIONS

The authors equally contributed to the preparation of this report.

## CONFLICT OF INTEREST STATEMENT

The authors declare no conflicts of interest.

## ETHICS STATEMENT

No ethic, nor patient statement.

## Data Availability

Data sharing not applicable to this article as no datasets were generated or analyzed during the current study.

